# Alterations of Pregnant Gait during Pregnancy and Post-Partum

**DOI:** 10.1038/s41598-018-20648-y

**Published:** 2018-02-02

**Authors:** Qichang Mei, Yaodong Gu, Justin Fernandez

**Affiliations:** 1 Faculty of Sports Science, Ningbo University, Ningbo, 315211 China; 2 Research Academy of Grand Health Interdisciplinary, Ningbo University, Ningbo, 315211 China; 3 Auckland Bioengineering Institute, University of Auckland, Auckland, 1010 New Zealand; 4 Department of Engineering Science, University of Auckland, Auckland, 1010 New Zealand

## Abstract

Physique changes during pregnancy lead to gait characteristic variations. This study aimed to analyse gait of pregnant individuals throughout pregnancy and post-partum. Sixteen healthy pregnant women volunteered as participants and had their lower limb kinematics analysed through a VICON three-dimensional motion system and plantar pressure measured with a Novel EMED force plate. Significant changes were observed in pelvic anterior motion, hip and ankle joint kinematics. Mean pressure distribution and COP trajectory deviation altered accordingly with increased pregnancy time, compared with post-partum. This longitudinal study of pregnant gait biomechanics in T2, T3 and PP reveals lower extremity kinematic and foot pressure alterations to adapt to pregnancy related changes, and the COP trajectory highlights a falling risk during pregnancy, particularly in T3.

## Introduction

Pregnancy is a normal condition experienced by many women that leads to anatomical and physiological alterations, affecting the musculoskeletal system and showing altered gait and posture characteristics. In the process of delivery, body weight increases greatly in the trunk (belly) during fetus growth, which leads to physique changes^1^. Previous studies showed that integrated changes during pregnancy increased falling risk for pregnant women^2–4^. The plantar loading would redistribute as the body weight increase and centre of mass relocate, which is reported to be correlated with foot complaints^5,6^. Thus, the motor system adjusts accordingly to compensate these changes to keep postural balance and gait stability^2–4,7–10^.

Previous investigation of non-pregnant and pregnant women in different trimesters presented altered lower extremity kinematics and kinetics^6,11–14^. A decreased step length and increased step width with slower walking velocity, and longer double-support time with progression of pregnant trimesters was the gait pattern observed for stability improvement^11,13,15,16^. The trunk and lower extremity motion changed greatly during pregnancy, particularly the pelvis and hip joint^16^. Branco *et al*.^17^ found that joint kinematics showed significant decrease of hip extension and adduction and ankle plantarflexion during pregnancy. These were mainly affected by fear of balance loss during late pregnancy^13^. Due to the constantly upward-transferred centre of gravity, the ability to control trunk equilibrium declined in late pregnancy^18^. Significant increases in mean pelvic and ankle separation widths and anterior tilt of the pelvis during pregnancy were also found^19^. The so-called “duck”, “penguin” or “waddle” pregnant gait may come into being due to significant variations of joints^18,20^.

Owing to the particular physique change to the belly, the lower extremity experienced significant changes during pregnancy^21^. Previous research showed the stiffness of the longitudinal foot arch significantly decreased during the first trimester and was not obvious during the last trimester^22^. Gaymer *et al*.^12^ reported that midfoot plantar pressure significantly increased during late pregnancy. This may provide an explanation for the decreased stability during pregnancy, and foot pronation is observed among pregnant individuals^5,21^. However, pregnant subjects typically adopt the strategy of increasing ankle stiffness to prevent falling^3^. The larger COP medial-lateral displacement is believed to be an adaptation strategy^23^. What else, the redistributed plantar pressure, especially to the forefoot regions, attributed to foot pain and other complaints^5,6,22^.

Pregnant women are at high risk of falling (28%)^24^, showing a greatly increased hospitalization rate from falling as trimesters progress (9.4% in the first, 11.3% in the second, and 79.3% in the third trimester)^2^. Pregnant women are 2.3 times more likely than non-pregnant or reproductive-aged women of falling^25^. These reported findings concerning pregnant gait alterations in different trimesters were all conducted during pregnancy. Apart from basic pregnant gait character analysis, the stair ascent and descent locomotion was investigated to check the ground reaction force alteration to reveal falling risks^26^. However, few studies have investigated gait biomechanical characteristic changes of pregnant individuals in different trimesters and post-partum, which lasts six weeks after delivery^2^.

This study took a longitudinal perspective to study pregnant women in the second trimester (**T2**), third trimester (**T3**) and 4-month post-partum (**PP**), integrating kinematics with kinetics analysis of normal pregnant walking to investigate gait characteristics. It is hypothesized that pregnant individuals would present altered gait characteristics throughout trimesters and post-partum, and this is measured via evaluation of foot pressure, pelvis and lower extremity kinematics.

## Results

**Figure 1:**
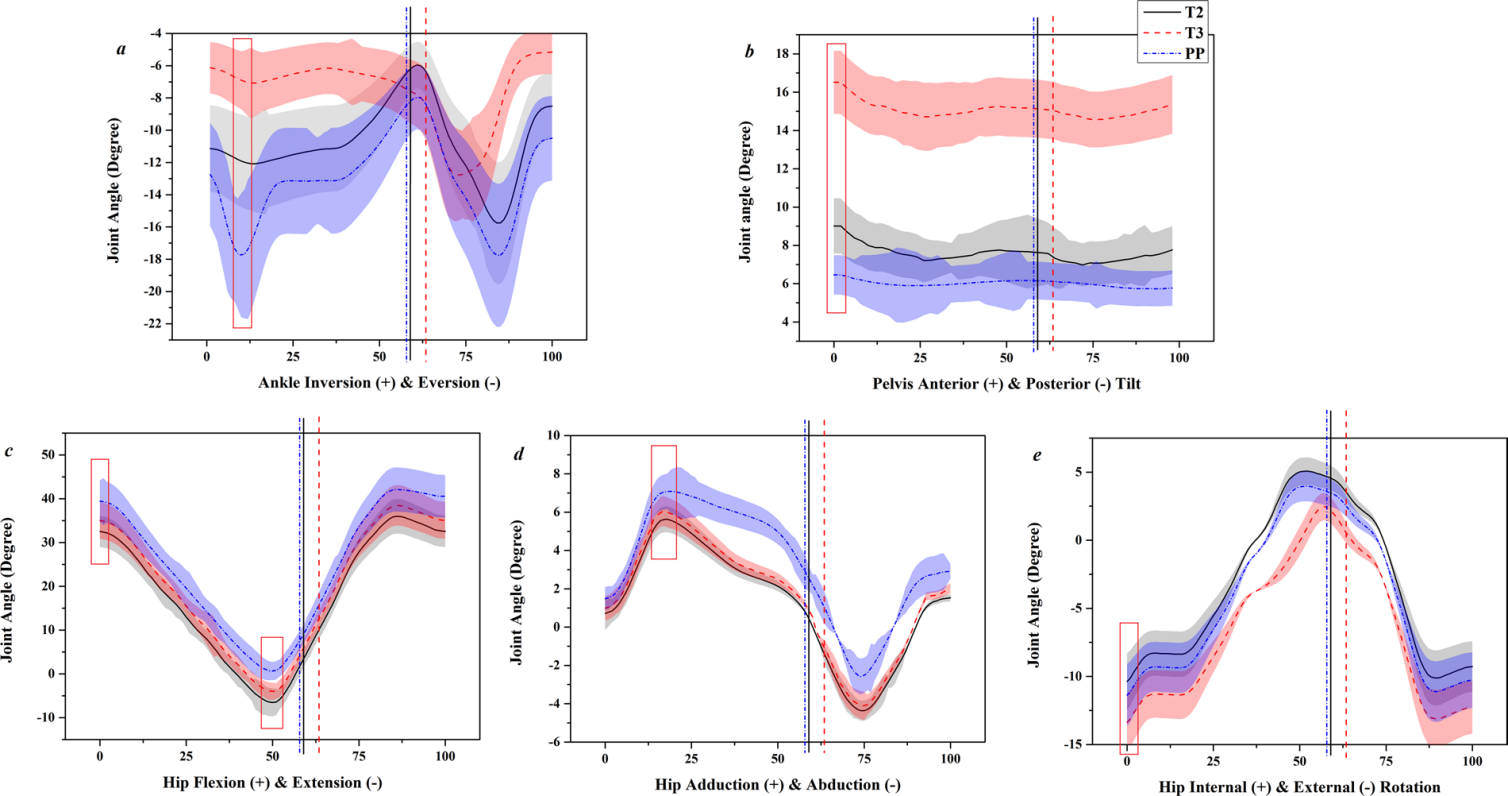
Mean angle curve (with SD) of ankle, pelvis and hip in a gait cycle (**T2**-solid black line, **T3**-dashed red line, and **PP**-dot blue line) with vertical lines indicating the stance phase and red rectangles highlighting peak value significance.

Based on the measured stride time and stance time, the stance phase (SP) are indicated in the joint angle figures with vertical solid line for T2 (58.95%), vertical dashed line for T3 (63.42%) and vertical dot line for PP (57.82%) respectively, to illustrate walking gait alterations. Lower extremity joints show consistent tendency in the stance phase, apart from significant inversion/eversion of ankle (Fig. 1a) and anterior tilt of pelvis (Fig. 1b) in T3. The other peak angle values with significance (*p* < 0.05) are highlighted with red rectangles (Fig. 1) and illustrated in Table 1.

**Table 1:** The peak angle values of ankle, pelvis and hip in the stance of T2, T3 and PP (degree, n = 16).

		T2	T3	PP
Ankle	Inversion	−7.28 ± 1.75	−6.13 ± 1.59	−8.02 ± 2.05
	Eversion	**−12.07 **±** 2.89↑**^**#**^	**−7.07 **±** 2.18↓**^**&**^	**−17.76 **± 4.02↑^*^
Pelvis	Anterior tilt	**8.46 **±** 2.09↓**^**#**^	**16.70 **±** 2.18↑**^**&**^	6.35 ± 3.02
Hip	Flexion	32.67 ± 2.58	**34.81 **±** 3.52↓**^**&**^	**39.2 **± 3.11↑^*^
	Extension	−4.13 ± 1.31	**−2.89 **±** 1.05↑**^**&**^	**1.14 **± 1.12↓^*^
	Adduction	5.13 ± 1.57	**6.20 **±** 2.06↓**^**&**^	**7.74 **± 1.66↑^*^
	Abduction	1.03 ± 1.87	−1.38 ± 1.31	2.98 ± 1.06
	Internal rotation	4.96 ± 1.96	2.58 ± 2.16	4.33 ± 1.65
	External rotation	**−11.04 **±** 3.74↓**^**#**^	**−14.05 **±** 3.96↑**^**&**^	−12.12 ± 4.02

Note: ^**#**^represents significance between T2 and T3, ^&^represents significance between T3 and PP, and ^*^represents significance between T2 and PP. Minus ‘−’represents position relative defined motion axis. Arrows (**↑/↓**) mean increase or decrease between two variables.

Peak ankle eversion angles significantly decrease from T2 to T3 (*p* = 0.021), but increases in PP compared with T2 (*p* = 0.029) and T3 (*p* = 0.006). The pelvic peak anterior tilt angle in T3 is greater than T2 (*p* = 0.013) and PP (*p* = 0.009). Peak hip flexion angle shows significance between comparison of PP with T2 (*p* = 0.023) and T3 (*p* = 0.031). Peak hip extension angle shows differences between PP with T2 (*p* = 0.046) and T3 (*p* = 0.031). PP have larger peak hip adduction angle in stance than T2 (*p* = 0.042) and T3 (*p* = 0.039). Peak external rotation angle in T3 is greater than T2 (*p* = 0.034) and PP (*p* = 0.036).

**Figure 2:**
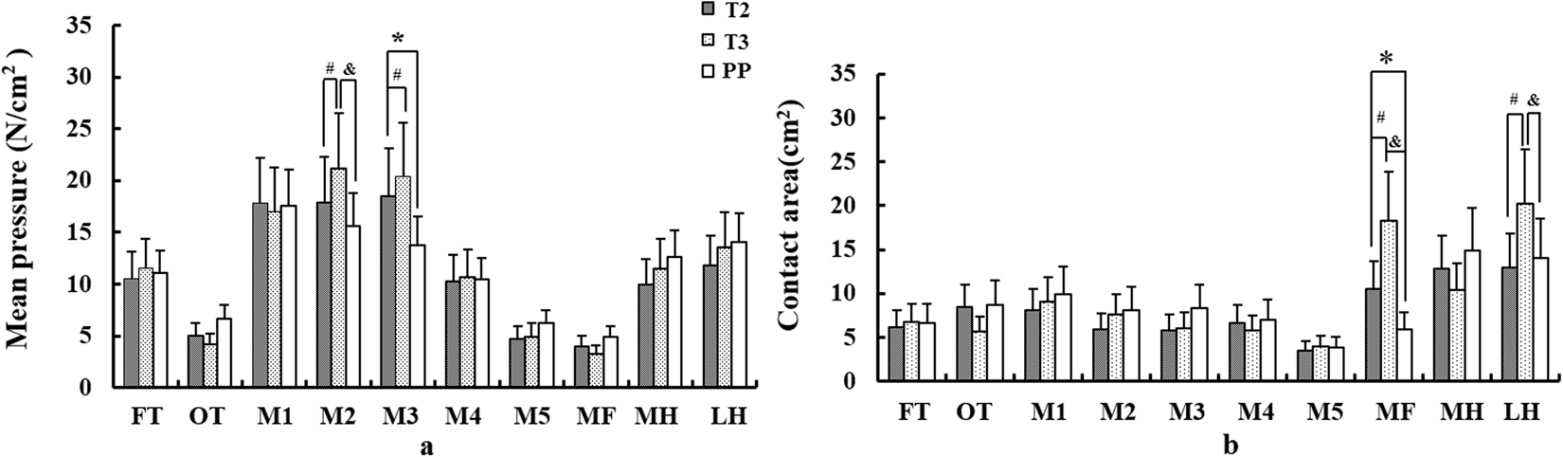
Comparison of mean pressure (**a**), contact area (**b**) among T2, T3 and PP. ^**#**^Represents significance between T2 and T3, ^**&**^represents significance between T3 and PP, and *represents significance between T2 and PP.

In the Fig. 2a, mean pressure in M2 of T3 are significantly larger than T2 (*p* = 0.038) and PP (*p* = 0.029). Mean pressure in T3 was greater than T2 (*p* = 0.039) and PP (*p* = 0.021) in M3. As the gestational weeks progressed, an increase in MF contact area is observed (*p* = 0.022 for T2 and T3), but significant reduction in PP (*p* = 0.025 for T2 and PP, *p* = 0.014 for T3 and PP). The T3 shows larger contact area than T2 (*p* = 0.042) and PP (*p* = 0.041) in LH (Fig. 2b).

**Figure 3:**
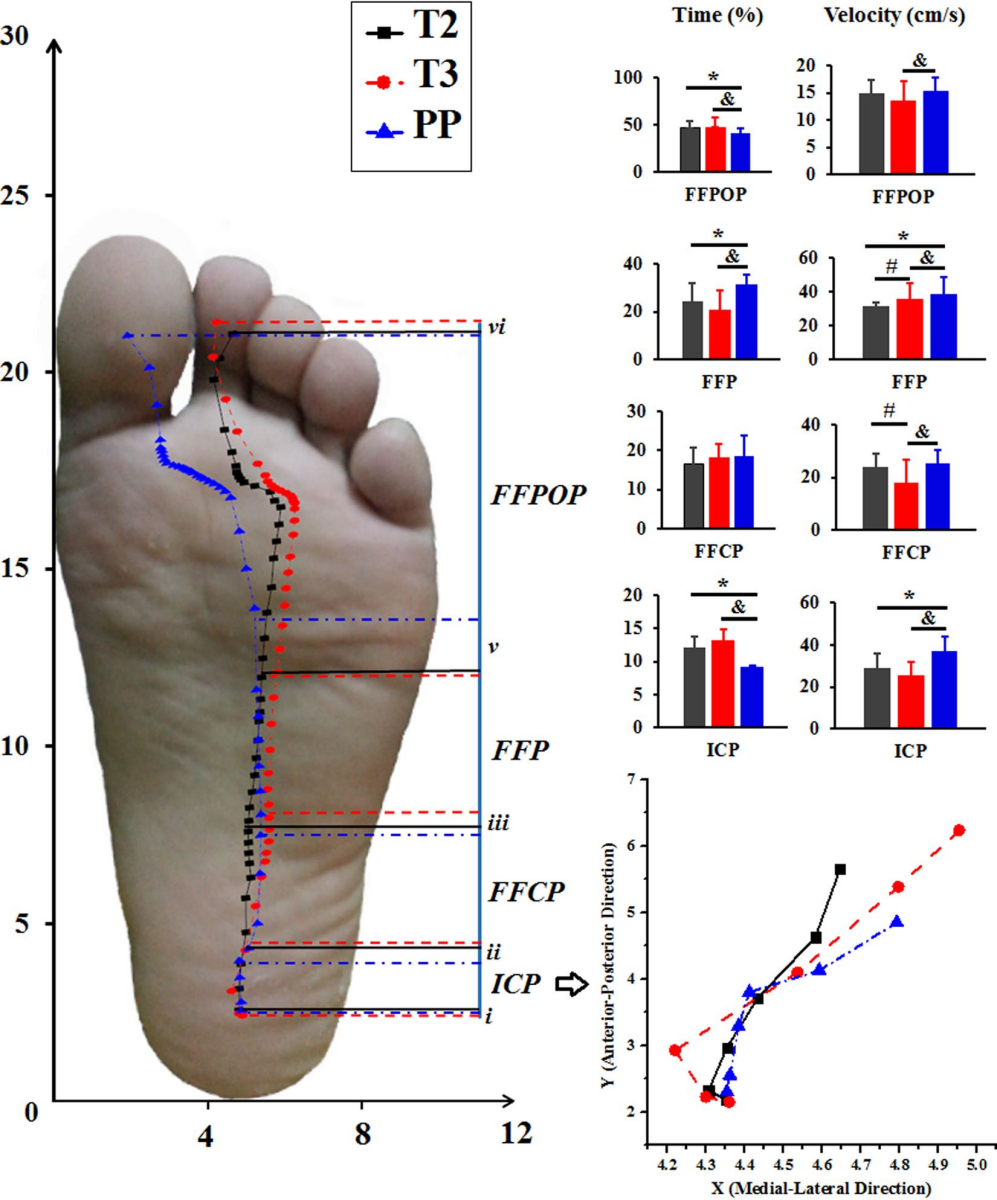
The illustration of COP trajectory, with indication of time (%) and velocity (cm/s), in different phases of stance. The solid black line, dashed red line and dot blue line respectively represents T2, T3 and PP. The ICP, FFCP, FFP and FFPOP means initial contact phase, forefoot contact phase, foot flat phase and forefoot push off phase, respectively. ^**#**^Represents significance between T2 and T3, ^**&**^represents significance between T3 and PP, and *represents significance between T2 and PP.

In Fig. 3, the COP trajectory presents a lateral shift in the ICP during T2 and T3 (with amplified illustration in the right corner), particularly the T3 with significantly larger average COP coordinates in the x-direction (***A***_***x***_) than T2 (*p* = 0.032) and PP (*p* = 0.033). The COP Deviation (D_n_) increase significantly in T3 compared with PP during the ICP (*p* = 0.038) (Table 2). However, in the FFCP, D_n_ of the T2 (*p* = 0.019) and T3 (*p* = 0.032) decreases greatly compared with PP. In FFP, there is significant increase of D_n_ in T2 (*p* = 0.018) and T3 (*p* = 0.028) compared with PP. A significant decrease in T3 compared with T2 (*p* = 0.036) was also exhibited. The ***A***_***x***_ in T2 and T3 of FFPOP increased compared with PP (*p* = 0.031, *p* = 0.029), with observable lateral transfer. The time (in percentage) of each phase in stance also shows significant difference. The T2 and T3 are significantly larger than PP, with p = 0.01 and 0.00 in the ICP. In the FFP, PP is longer than T2 (p = 0.023) and T3 (0.015). PP has shorter FFPOP time comparing with T2 (p = 0.032) and T3 (p = 0.03). As for the velocity in each phase, PP is significantly faster than T2 (p = 0.039) and T3 (P = 0.009) in the ICP. T3 presents smaller velocity than T2 (p = 0.024) and PP (p = 0.022) in the FFCP. The T2 has slower velocity in FFP than T3 (p = 0.038) and PP (p = 0.026), and T3 is also slower than PP (p = 0.042). In the FFPOP, PP shows significantly faster velocity than T3 (p = 0.028).

**Table 2:** The averaged COP coordinates (A_n_) and Deviation (D_n_) in four phases of stance.

		T2	T3	PP
ICP	***A***_***x***_	**4.36↓** ^**#**^	**4.52↑** ^**&**^	4.35
	***A***_***y***_	3.57	3.83	3.49
	D	1.13 ± 0.63	**1.42 ± 0.57↑** ^**&**^	0.78 ± 0.51
FFCP	***A***_***x***_	5.49	6.01	5.1
	***A***_***y***_	8.88	9.61	11.31
	D	1.57 ± 1.31	**1.97 ± 0.95↓** ^**&**^	3.04 ± 1.67*
FFP	***A***_***x***_	6.23	6.79	4.7
	***A***_***y***_	15.69	16.27	18.42
	D	**1.53 ± 0.74↑** ^**#**^	**1 ± 0.49↑** ^**&**^	**0.26 ± 0.15↓** ^*^
FFPOP	***A***_***x***_	5.85	**6.4↑** ^**&**^	**4.15↓** ^*^
	***A***_***y***_	19.66	19.1	20.05
	D	1.41 ± 0.81	1.56 ± 0.72	1.25 ± 0.46

Note: The ***C***_***xn***_ and ***C***_***yn***_ are constant centre of pressure trajectory coordinates in the medial-lateral direction (***x***) and anterior-posterior direction (***y***), and the ***A***_***xn***_ and ***A***_***yn***_ are averaged centre of pressure coordinates in the medial-lateral direction (***x***) and anterior-posterior direction (***y***). ^**#**^Represents significance between T2 and T3, ^**&**^represents significance between, T3 and PP, and *represents significance between T2 and PP. Arrows (**↑/↓**) mean increase or decrease between two variables.

## Discussion

Pregnant women have been found to exhibit special gait characteristics compared with non-pregnant women in previous studies. This study aimed to analyse the gait biomechanics of pregnant women throughout pregnancy and post-partum from a longitudinal perspective.

Contrasting the smaller inversion-eversion motion range during stance (Fig. 1a) of this study with previous research^17^, we observed a smaller peak ankle eversion in T3 than both T2 and PP. This is consistent with a previously reported strategy of increased ankle stiffness (which leads to a smaller peak ankle eversion) to enhance postural control and gait stability^3^. Pelvic anterior tilt increased with trimester progression with the growth of fetus and belly^17,19^. The decreased hip flexion and extension has been linked with reduced step length, and the decreased hip abduction and increased hip external rotation are related with larger step width^11,17,19,20^. These alterations may attribute to the biomechanical mechanism, which pregnant women adopt for motor stability maintenance and prevention of falling.

Mean pressure in M2 and M3 significantly increased from T2 to T3, but reduced in PP. This findings showed consistency with the central shift of the COP in the forefoot (Fig. 3), which was reported with forward shift of plantar loading during pregnancy^5,6^. The MF contact area increased from T2 to T3 but decreased in PP, which was consistent with a previous report of pregnant vs non-pregnant women^12^. One possible explanation is related to foot morphology changes^22,27^, although participants in this study with abnormal changes were excluded by gynaecologists. Another explanation may be the foot longitudinal arch drop^5,22^ and foot stiffness^3^ from increased body weight as pregnancy progression.

The COP trajectory indicates gait stability during stance^17,22,28^. The gait dynamics could be inferred from the COP deviation (D_n_). As D_n_ increased, COP dispersed greatly, indicating declined gait stability. The significantly increased D_n_ in T2 and T3 could be indicators of reduced gait stability and increased falling risk^17,22^, particularly in the FFP. In the ICP, D_n_ of T3 deviated significantly lateral (highlighted in the right corner of Fig. 3), showed greater D_n_ and averaged ***A***_***x***_ (Table 2), which may result from motor adapted shock absorption owing to greatly increased body weight (with fetus)^28^. In the FFCP, the D_n_ was higher during PP, which may be related with recovered stance characteristics with non-pregnant walking^7,22^. The D_n_ in T2 and T3 was consistent with COP changes in FFP and FFPOP. Specifically, T2 and T3 presented significantly larger ***A***_***x***_ than PP, which may be indicators of laterally shifted COP and altered walking stance stability characteristics^2,3,9^. The amplified medial shift of COP in ICP of T3 may be linked with foot pronation, which was believed to be a shock absorption strategy for landing stability and safety^21^. Further, the lengthened time and shortened velocity during pregnancy of ICP is key evidence in this study. In the FFCP and FFP, the presented COP deviation could be explained with foot rigid structure, such as the dropped foot longitudinal arch due to increased body weight during pregnancy^3,10^. ICP of one foot occurs with FFPOP of another foot during walking (double support), both phases are closely related with gait stability (landing stability and pushing-off stability). The lengthened time and reduced velocity in ICP and FFPOP are believed to be the motion adaptation strategy for gait stability, which would also result in reduced FFCP and FFP time as compensation. Compared the COP time and velocity of the PP period in this study with previous reports of non-pregnant women^17,21,22,29^, it was revealed that the COP trajectory was similar in both.

What deserves special attention in this study is that pregnant participants in (4 months) post-partum present similar lower extremity kinematic and COP trajectory characteristics compared with non-pregnant participants, compared with previous findings^17,21,23^. This reveals that pregnant individuals may own flexible motion capacity to adapt pregnancy-related alterations. Based on this finding, exercise protocols could be formulated^1,30^ in future studies, to reduce incidences resulting from pregnancy^25,31^. One limitation of this study is the lack of gait biomechanics data in the first trimester as most women are unavailable for study via the hospital in this period due to nausea. Another limitation concerns the reversible or non-reversible changes in pregnant foot shape, which might affect plantar loading and need further analysis via collecting larger data sets throughout pregnancy and post-partum.

In conclusion, this longitudinal study of pregnant gait biomechanics in T2, T3 and PP reveals lower extremity kinematic and foot pressure alterations to adapt to pregnancy related changes, and the COP trajectory highlights a falling risk during pregnancy, particularly in T3.

## Methods

### Participants

Sixteen healthy pregnant women (age: 32.5 ± 3.64years and height: 161.8 ± 5.27 cm) participated in the study during three stages, including the second trimester (**T2**) (21 ± 0.58 gestational weeks and weight: 57.4 ± 2.03 kg), third trimester (**T3**) (29 ± 0.72 gestational weeks and weight: 59.6 ± 2.36 kg) and 4-month post-partum (**PP**) (weight: 51.8 ± 2.21 kg), respectively. The averaged foot length and width are 228 mm and 105 mm throughout pregnancy and post-partum. This study with detailed guidelines for participants’ safety and experiment protocols was approved by the Human Ethics Committee of Ningbo University (ARGH20150616), and all methods were performed in accordance with the guidelines and regulations. Prior to the test, all subjects gave informed consent knowing test procedures and requirements. All subjects presented with no injury or pain to the lower limb or foot deformities throughout the test process.

### Experiment Protocols

All participants walked barefoot on a 10-meter walkway at their self-selected comfortable speed to present normal gait characters, with right foot striking on the force plate. Subjects were instructed to perform five minutes’ walking for warm up, lab environment familiarization and step adjustment. A gait cycle was defined as ipsilateral heel (right foot in this study) contact ground twice. Each participant conducted five trials of walking test with synchronous collection of kinematics and plantar pressure data, which were used to obtain averaged values to minimise inter-trial errors. The collected kinematics data for analysis in this study include peak angle values in a stance and joint angle curves during a gait cycle.

An eight-camera three-dimensional motion analysis system (VICON Motion System Ltd., Oxford, England) was used to capture kinematics of the pelvis, hip, knee and ankle with a frequency of 200 Hz. The standard Plug-In Gait model with 16 reflective markers was used to define joint centre and motion axes, with marker-set locations including: anterior-superior iliac spine (Left and Right ASIS), posterior-superior iliac spine (Left and Right PSIS), lateral thigh (Left and Right THI), lateral knee (Left and Right KNE), lateral tibia (Left and Right TIB), lateral ankle (Left and Right ANK), toe (Left and Right TOE) and heel (Left and Right HEE). With the growth of fetus and belly, which could influence the location of left and right ASIS, one professional obstetrics doctor assisted locating the anatomical position during the gait test to ensure the accuracy of marker placement and pregnant participants’ safety. Peak joints (pelvis, hip and ankle) angles in a stance phase were utilised for analysis, including pelvis anterior/posterior tilt, flexion/extension (dorsiflexion/plantarflexion for ankle) in the sagittal plane, abduction/adduction (inversion/eversion for ankle) in the coronal plane, and internal/external rotation in the horizontal plane^16,17^.

A Novel EMED force plate (Novel GmbH, Munich, Germany) was fixed in the middle of the walkway to record plantar loading with a frequency of 100 Hz. The foot was divided into ten anatomic parts, including first toe (FT), 2^nd^ to 5^th^ or other toes (OT), first metatarsal (M1), second metatarsal (M2), third metatarsal (M3), fourth metatarsal (M4), fifth metatarsal (M5), middle foot (MF), medial heel (MH), and lateral heel (LH). Variables included mean pressure (averaged pressure distributed to each region measures from the force) and contact area (the contact area of plantar surface with the force plate)^6,12,32^ for above ten parts. Total contact area was defined as the number of EMED force plate sensors activated (with each sensor having a known area). The centre of pressure trajectory was analysed with further spatial details outline below.

**Figure 4:**
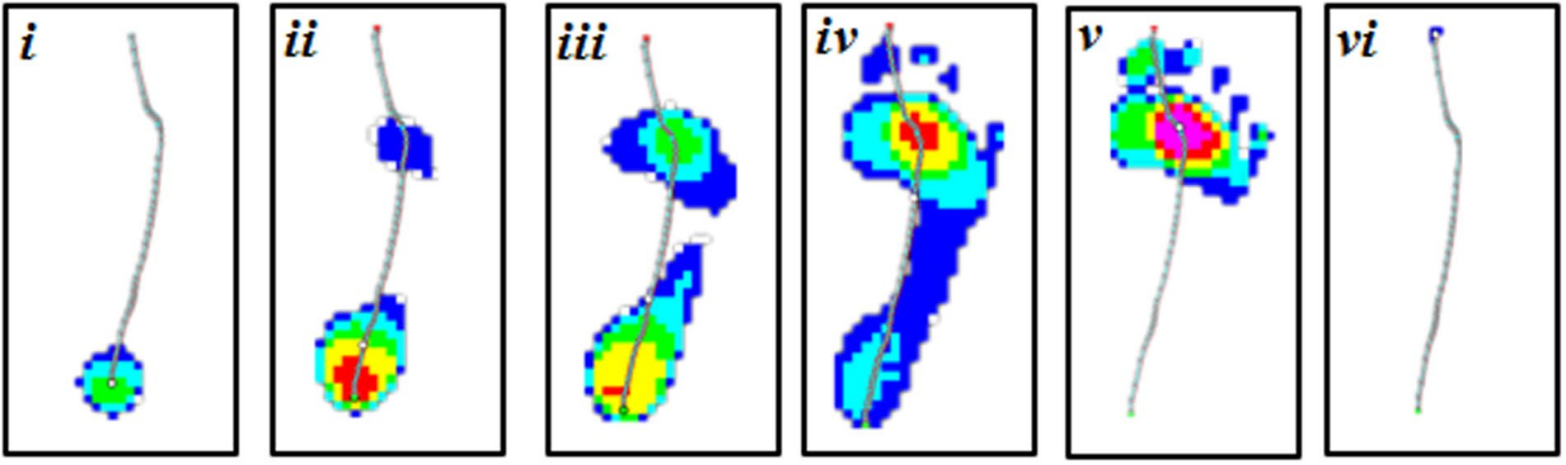
The illustration of cut-off points (***i*** to ***vi***) for stance phase division. (***i***, heel first contact; ***ii***, one of the metatarsals contact; ***iii***, all metatarsals head contact; ***iv***, full foot contact; ***v***, heel off; ***vi***, toes off).

The stance (100%) of the right foot was divided into four phases including six cut- off points (from ***i*** to ***vi*** in the Fig. 4) based on previous studies^28,33,34^. The initial contact phase (**ICP**) begins from heel first contact (***i***) to one of the metatarsals contact (***ii***) the plate. The forefoot contact phase (**FFCP**) begins from one of the metatarsals contact (***ii***) to all metatarsals head contact (***iii***) the plate. The foot flat phase (**FFP**) begins from all metatarsals contact (***iii***), full foot contact (***iv***) to heel off (***v***) the plate. And the forefoot push off phase (**FFPOP**) begins from heel off (***v***) to toes off (***vi***) the plate.

The COP trajectory is resolved into coordinates x and y, which are normalized to feet width in the medial-lateral direction (x) and length in the anterior-posterior direction (y). ***C***_***xn***_ and ***C***_***yn***_ are variables referring to the coordinates of the centre of pressure trajectory in the medial-lateral direction (***x***) and anterior-posterior direction (***y***), respectively. ***A***_***xn***_ and ***A***_***yn***_ are variables referring to the averaged coordinates of the centre of pressure in the medial-lateral direction (***x***) and anterior-posterior direction (***y***), respectively.

The deviation of COP trajectory, ***D***_***n***_ was calculated with following equation^35^:

$${D}_{n}=\sqrt{{({C}_{xn}-{A}_{xn})}^{2}+{({C}_{yn}-{A}_{yn})}^{2}}$$

The phase time (in percentage) and velocity (in cm/s), averaged COP coordinates (***Ax & Ay***), and deviation (***D***) of collected data in second trimester (**T2**), third trimester (**T3**) and post-partum (**PP**) were taken to analyse and illustrate gait characteristics.

### Statistical analysis

The SPSS 19.0 (SPSS Inc., Chicago, IL, USA) were utilized for statistical analysis. The kinematic and plantar pressure variables (peak angle values, mean pressure, contact area, phase duration and velocity, averaged coordinates and D) was checked and confirmed with the Shapiro-Wilk normality test. For the T2, T3 and PP data paired analysis, the Repeated Measures ANOVA analysis were conducted to check the significance of each viable. The trial to trial reliability of COP coordinates in medial-lateral (***x***) and anterior-posterior (***y***) directions was tested with intra-class correlation coefficients (ICC), all showing good reliability (>0.75)^21,22^. All the significance level was set at 0.05.
